# Supplementary material for: Evaluation of the methane paradox in four adjacent pre-alpine lakes across a trophic gradient
Source: Nat Commun. 2023 Apr 15;14:2165. doi: 10.1038/s41467-023-37861-7 (PMC10105773; doi:10.1038/s41467-023-37861-7)
Supplement: Supplementary file 1 — Supplementary Information [file 41467_2023_37861_MOESM1_ESM.pdf]

Supplementary Information for

Evaluation of the methane paradox in four adjacent pre-alpine lakes across a trophic gradient

César Ordóñez[1*], Tonya DelSontro[1, 2*], Timon Langenegger[1], Daphne Donis[1], Ena L. Suarez[1], and Daniel F. McGinnis[1*]

[1]Aquatic Physics Group, Department F.-A. Forel for Environmental and Aquatic Sciences (DEFSE), Faculty of Science, University of Geneva, Uni Carl Vogt, 66 Boulevard Carl-Vogt, 1211 Geneva, Switzerland

[2]Now at Department of Earth and Environmental Sciences, University of Waterloo, Ontario, Canada

[*]Corresponding authors: cesar.ordonez@unige.ch, tonya.delsontro@uwaterloo.ca, daniel.mcginnis@unige.ch

Supplementary

Supplementary Tables

Supplementary Table 1. Study sites main characteristics.

Lake	Latitude (°N)	Longitude (°E)	Altitude (m.a.s.l)	Max. Depth (m)	Surf. Area (ha)
Bretaye	46.326	7.072	1785	8	4
Chavonnes	46.333	7.085	1692	25	5
Lioson	46.386	7.128	1848	28	7
Noir	46.327	7.079	1715	9	1

Supplementary Table 2. Trophic state classification for each lake based on the trophic state index (TSI) based on water column average of total phosphorus (TP), surface chlorophyll-a concentration (Chl$a$) and Secchi disk depth (Z_s) for June 2018 following Carlson[1].

Lake	TSI(TP)	TSI (Z_s)	TSI(Chl$a$)	Avg. TSI ± SD	Trophic State
Bretaye	55	41	65	54 ± 12	Eutrophic
Chavonnes	34	38	58	44 ± 13	Mesotrophic
Lioson	26	28	59	38 ± 18	Oligotrophic
Noir	36	45	65	49 ± 15	Eutro/Mesotrophic

Supplementary Table 3. Comparison between average stable carbon isotopes of CH_4 ($\delta^{13}C_{CH_4}$) at the surface water and $\delta^{13}C_{CH_4}$ at the top and bottom of the porewater measurement at each lake.

Lake	Date	Surface Water	Sediment	
		$\delta^{13}C_{CH_4}$ (‰)	$\delta^{13}C_{CH_4top}$ (‰)	$\delta^{13}C_{CH_4bottom}$ (‰)
Bretaye	June 2018	-52.0		
	July 2019	-48.8	-66.0	-66.5
	Sept 2018	-38.8	-48.4	-65.7
Chavonnes	June 2018	-62.3		
	July 2019	-61.2	-62.0	-60.0
	Sept 2018	-62.4		
Lioson	June 2018	-50.9		
	July 2019	-54.0		
	Sept 2018	-50.1		
Noir	June 2018	-54.5		
	July 2019	-49.9	-63.9	-65.9
	Sept 2018	-45.5	-66.2	-71.6

Supplementary Table 4. Summary of diffusive sediment CH_4 fluxes (F_s) and its associated sampling depth, temperature of overlying water (T_w), type of measurement (PW: Porewater and BC: benthic chamber) and zone classification.

Lake	Date	Depth (m)	Zone	T_w (°C)	Core location	Type	F_s (mmol m^{-2} d^{-1})
Bretaye	16/06/18	9	Deep	4.8	M1	PW	5.25
	16/06/18	5.2	Deep	4.8	C1	PW	1.53
	02/09/18	9	Deep	4.8	M1	PW	5.23
	02/09/18	3.5	Shallow	15.3	C1	PW	5.76
	20/07/19	8.4	Deep	8.8	M1	PW	12.6
	20/07/19	2.7	Shallow	19.7	C1	PW	0.50
	20/07/19	1.7	Shallow	19.7	C3	BC	12.65
	20/07/19	2.6	Shallow	19.7	C2	BC	12.48
Noir	20/06/18	10.9	Deep	4.5	M1	PW	8.54
	20/06/18	5.5	Deep	5.6	C1	PW	0.22
	03/09/18	10.5	Deep	5.4	M1	PW	6.54
	03/09/18	4.7	Shallow	13.5	C1	PW	2.18
	24/07/19	0.75	Shallow	22.3	ClitB	PW	2.94
	24/07/19	0.75	Shallow	22.3	ClitB	BC	1.62
	24/07/19	0.5	Shallow	22.3	C2	BC	1.33
	24/07/19	1	Shallow	22.3	C3	BC	1.33
	25/07/19	8	Deep	4.4	M1	PW	5.62
Chavonnes	23/07/19	0.5	Shallow	21.2	C1	PW	0.01
	23/07/19	0.6	Shallow	21.2	C1	BC	0.82
	23/07/19	0.5	Shallow	21.2	C2	BC	0.39
Lioson	23/07/19	0.2	Shallow	14.7	C1	BC	0.31
	23/07/19	0.3	Shallow	14.7	C2	BC	0.16
	23/07/19	0.25	Shallow	14.7	C3	BC	0.27

Supplementary Table 5. CH_4 ebullition flux needed ($F_{eb,need}$) to compensate the net production rate of CH_4 (P_{net}) in the surface mixed layer (SML) for June 2018 in Lac de Bretaye (BRE) and Lac Noir (NOI). The percentage of the ebullition flux that is dissolved in the SML (β) was estimated using McGinnis et al.[5]'s assuming a bubble diameter of 5 mm.

Lake	β (%)	$F_{eb,need}$ (mmol m^{-2} d^{-1})
BRE	5	44.5
NOI	3	429

Supplementary Table 6. Total (TP) and dissolved phosphorus (DP), dissolved silica (DSIL), dissolved inorganic nitrogen as nitrate plus nitrite (DIN) and total carbon (TC) measurements in the hypolimnion and epilimnion. The depth column describe the range of depth from/to the samples were integrated (Methods). No value (-) and below detection measurements (BD).

Lake	Date	Region	Depth (m)	TP ($mg\,m^{-3}$)	DP ($mg\,m^{-3}$)	DIN ($g\,m^{-3}$)	DSIL ($g\,m^{-3}$)	TC ($g\,m^{-3}$)
Bretaye	Jun-18	Epilimnion	0 to 4	20.7	9.0	0.02	0.39	19.73
		Hypolimnion	5 to 8	66.7	18.7	0.01	1.89	26.72
	Jul-19	Epilimnion	0 to 5.5	33.0	-	0.00	0.83	29.13
		Hypolimnion	6 to 8.5	672.0	191.0	0.02	7.36	42.52
	Sept-18	Epilimnion	0 to 5	10.0	7.3	0.03	2.77	20.16
		Hypolimnion	5.5 to 7.5	150.0	39.3	0.02	5.29	21.00
Noir	Jun-18	Epilimnion	0 to 5	4.0	2.3	0.02	1.02	29.52
		Hypolimnion	6 to 9.2	29.7	-	0.05	3.99	42.43
	Jul-19	Epilimnion	0 to 5	8.0	BD	BD	1.22	33.11
		Hypolimnion	6 to 10	57.0	5.0	0.02	8.21	56.48
	Sept-18	Epilimnion	0 to 5	3.7	2.7	0.03	3.21	19.03
		Hypolimnion	6 to 9	13.0	2.3	0.05	7.60	51.69
Chavonnes	Jun-18	Epilimnion	0 to 15	4.3	2.0	0.24	1.10	21.22
		Hypolimnion	18 to 26	26.3	3.0	0.18	2.61	23.99
	Jul-19	Epilimnion	0 to 8	BD	BD	0.19	0.67	20.77
		Hypolimnion	10 to 25	9.0	BD	0.15	3.37	25.10
	Sept-18	Epilimnion	0 to 10	2.7	1.0	0.17	1.70	15.91
		Hypolimnion	12 to 22	10.3	1.7	0.06	4.06	28.40
Lioson	Jun-18	Epilimnion	0 to 9	3.3	2.0	0.13	1.82	23.03
		Hypolimnion	13 to 25	5.0	2.7	0.16	2.55	24.05
	Jul-19	Epilimnion	0 to 14	BD	BD	0.07	1.63	22.62
		Hypolimnion	16 to 28	BD	BD	0.10	2.71	24.47
	Sept-18	Epilimnion	0 to 14	3.3	1.0	0.05	1.42	7.66
		Hypolimnion	16 to 26	2.7	2.0	0.08	3.26	10.33

Supplementary Table 7. Volumes and areas used in the full-scale mass balance model. A_z, A_a, A_s are the planar area at the bottom of the surface mixed layer, the surface area and sediment area respectively. $\forall_{SML}$ is the volume of the surface mixed layer (SML). L is the length scale used to estimate the horizontal dispersion coefficient for the lateral transport model.

Lake	Date	A_z (m^2)	A_a (m^2)	A_s (m^2)	$\forall_{SML}$ (m^3)	L (m)
Bretaye	Jun 2018	36443	45449	8688	49380	120.28
	Sept 2018	20609	45449	24820	165770	120.28
	July 2019	32832	45449	12552	83425	120.28
Noir	Jun 2018	8012	9960	2026	7509	56.31
	Sept 2018	3057	9960	7139	30778	56.21
	July 2019	6566	9960	3511	14063	56.31
Chavonnes	Jun 2018	54267	60311	5700	71935	138.55
	Sept 2018	28949	44233	14958	166037	118.65
	July 2019	47681	54716	8236	97588	131.97
Lioson	Jun 2018	64659	70052	5129	31557	149.32
	Sept 2018	43222	70052	27800	295316	149.32
	July 2019	57722	70052	12385	89918	149.32

Supplementary Figures

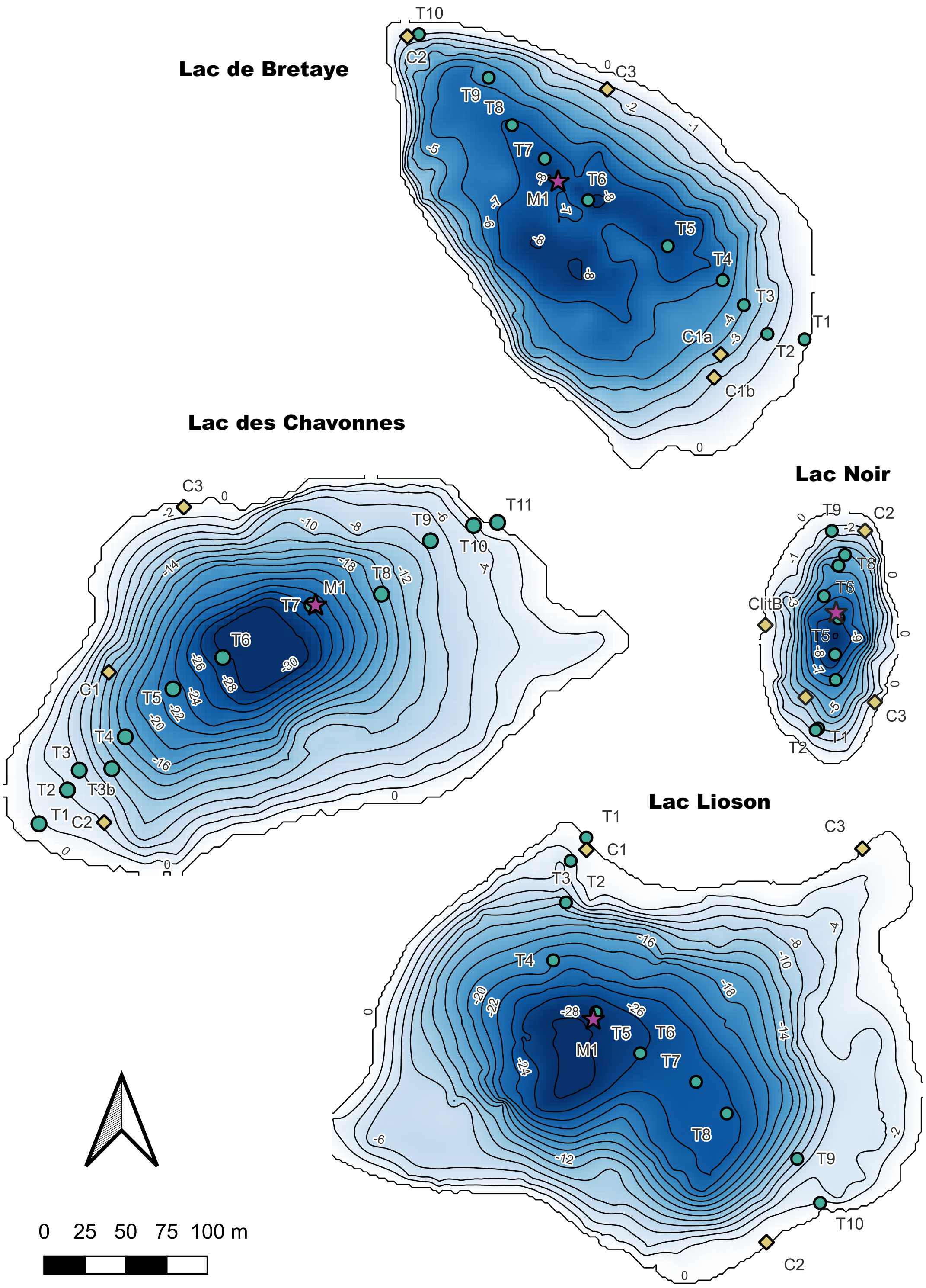

Supplementary Figure 1. Lake bathymetries obtained from sonar survey, sampling locations for each transect (T#; circles) and sediment sampling points (C#; diamond). M1 (star) is the location where water column profiles (temperature, oxygen, CH_4 concentrations and the stable carbon isotopes of CH_4) and nutrient sampling were performed. This map was created using sonar transects, and visually processed using Surfer® (Golden Software, LCC) and the Free and Open Source QGIS (Methods).

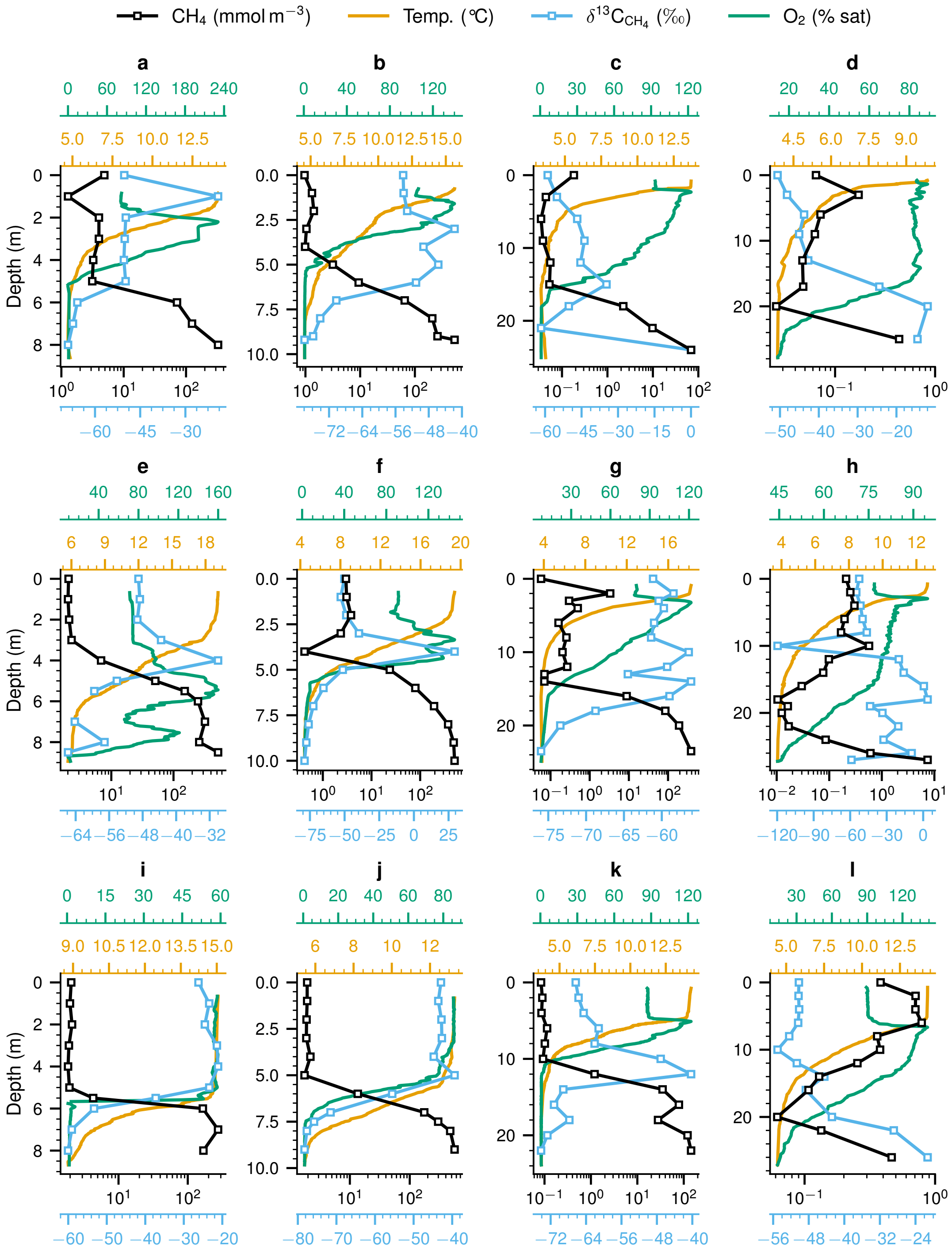

Supplementary Figure 2. Water column profiles of CH_4 concentrations (CH_4), temperature (Temp.), stable carbon isotopes of CH_4 ($\delta^{13}C_{CH_4}$) and dissolved oxygen saturation (O_2). For top to bottom **a-d** June 2018, **e-h** July 2019 and **i-l** Sept 2018 for left to right **a, e, i** Lac de Bretaye (BRE), **b, f, j** Lac Noir (NOI), **c, g, k** Lac des Chavonnes (CHA) and **d, h, l** Lac Lioson (LIO).

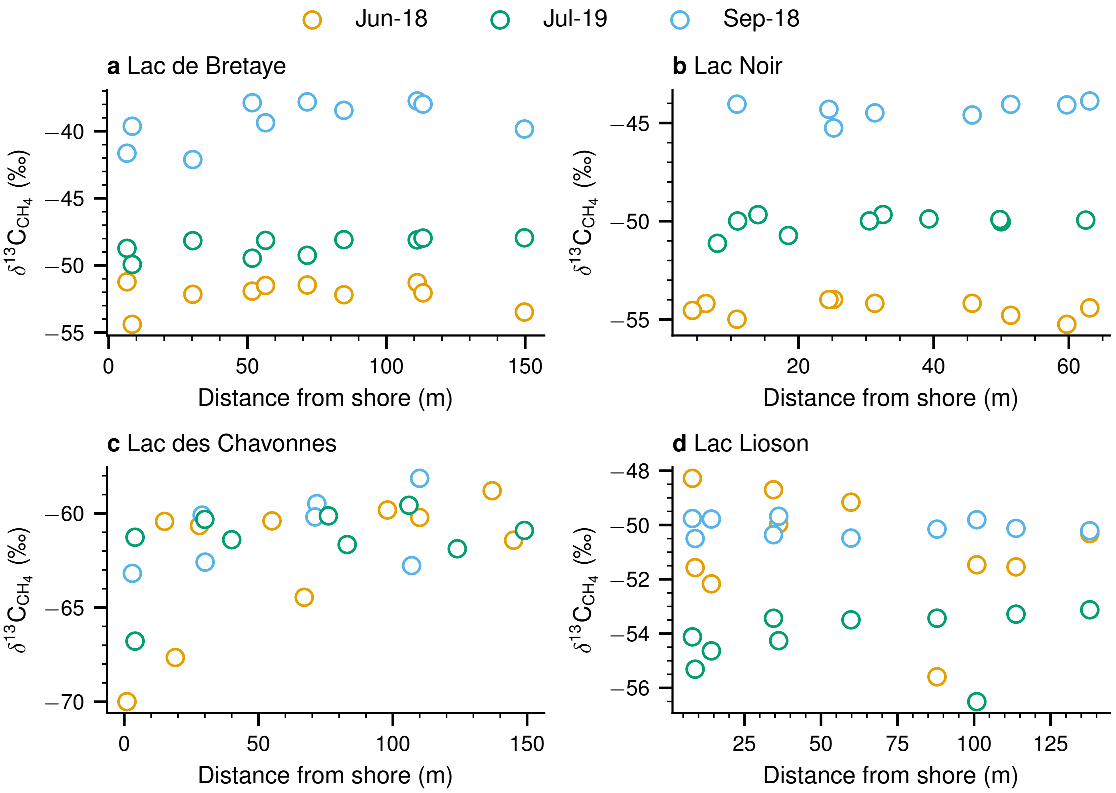

Supplementary Figure 3. Surface stable carbon isotopes of CH_4 ($\delta^{13}C_{CH_4}$) along the transects from shore to the center of **a** Lac de Bretaye, **b** Lac Noir, **c** Lac des Chavonnes and **d** Lac Lioson.

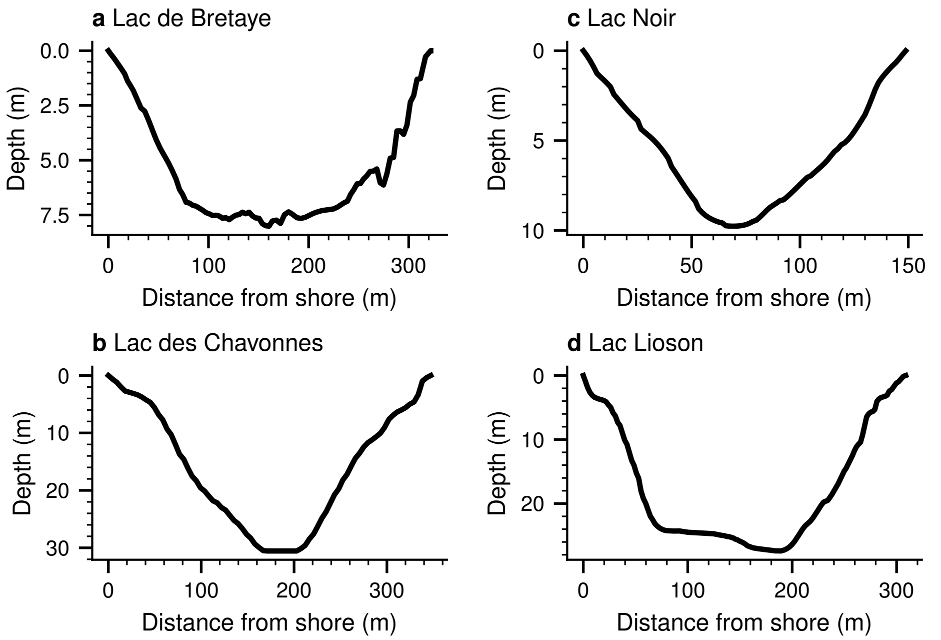

Supplementary Figure 4. Bathymetry profile along each transect in **a** Lac de Bretaye, **b** Lac Noir, **c** Lac des Chavonnes and **d** Lac Lioson.

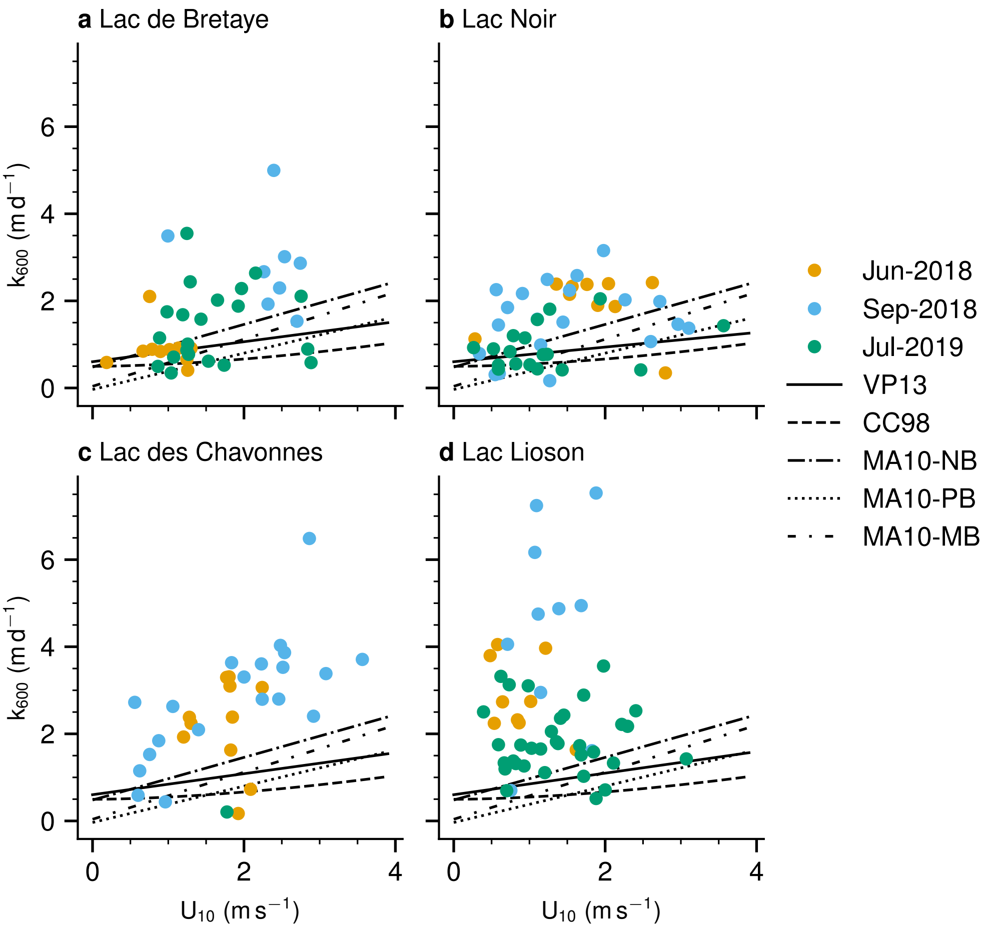

Supplementary Figure 5. Comparison between chamber-based k_{600} with different wind based parametrizations (CC98: Cole & Caraco[2]; MA10-NB, MA10-PB and MA10-MB: MacIntyre et al.[3] Negative Buoyancy, Positive Buoyancy and Mixed Model; VP13: Vachon & Prairie[4]). U_{10} is the wind velocity at 10 m in **a** Lac de Bretaye, **b** Lac Noir, **c** Lac des Chavonnes and **d** Lac Lioson.

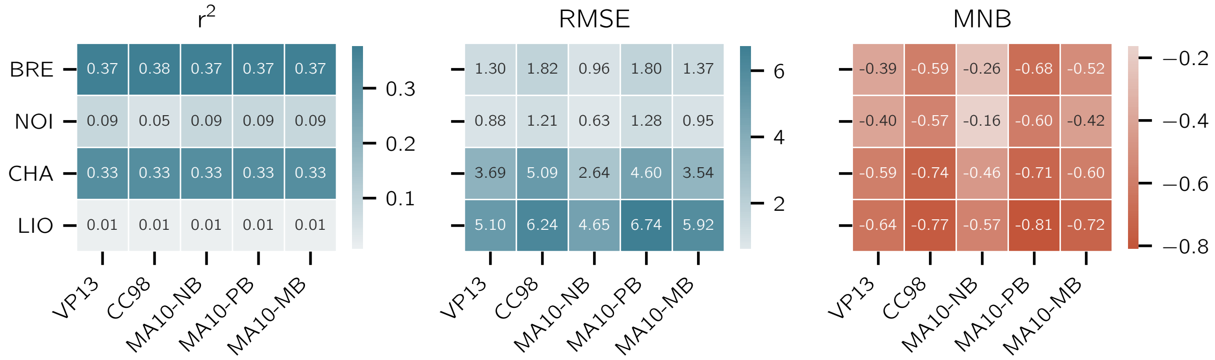

Supplementary Figure 6. Statistics between chamber based mass transfer coefficient and literature parameterizations (CC98: Cole & Caraco[2]; MA10-NB, MA10-PB and MA10-MB: MacIntyre et al.[3] Negative Buoyancy, Positive Buoyancy and Mixed Model; VP13: Vachon & Prairie[4]). R-square (r^2), root mean square error (RMSE) and mean normalized bias (MNB). Lac de Bretaye (BRE), Lac Noir (NOI), Lac des Chavonnes (CHA) and Lac Lioson (LIO).

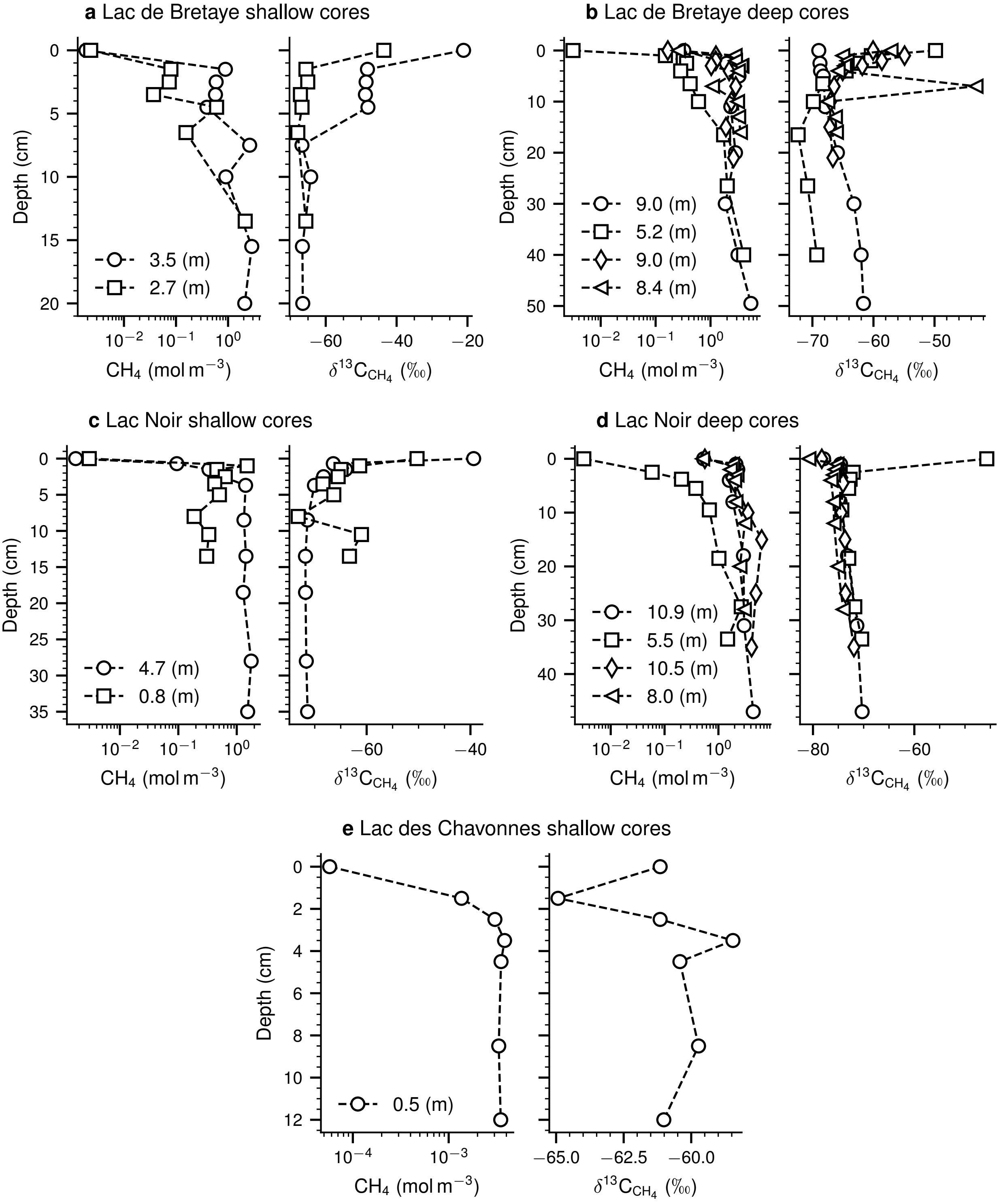

Supplementary Figure 7. Porewater CH_4 concentration (log scale) and stable carbon isotopes of CH_4 ($\delta^{13}C_{CH_4}$) profiles for Lac de Bretaye (**a** and **b**, deep and shallow cores respectively), Lac Noir (**c** and **d**, deep and shallow cores respectively) and **e** Lac des Chavonnes in a shallow core.

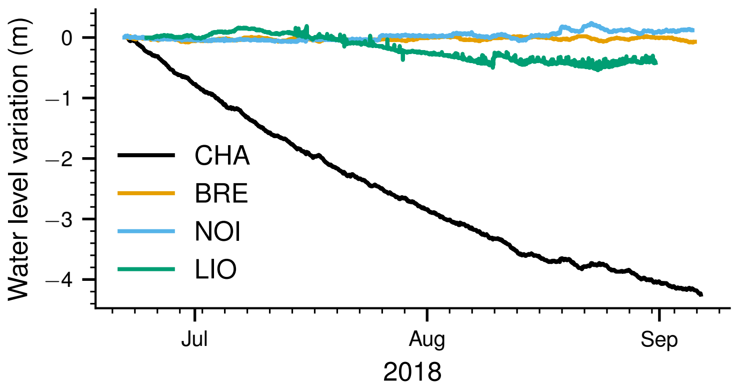

Supplementary Figure 8. Water level variations from the end of June to middle September of 2018 for Lac de Bretaye (BRE), Lac Noir (NOI), Lac des Chavonnes (CHA), Lac Lioson (LIO).

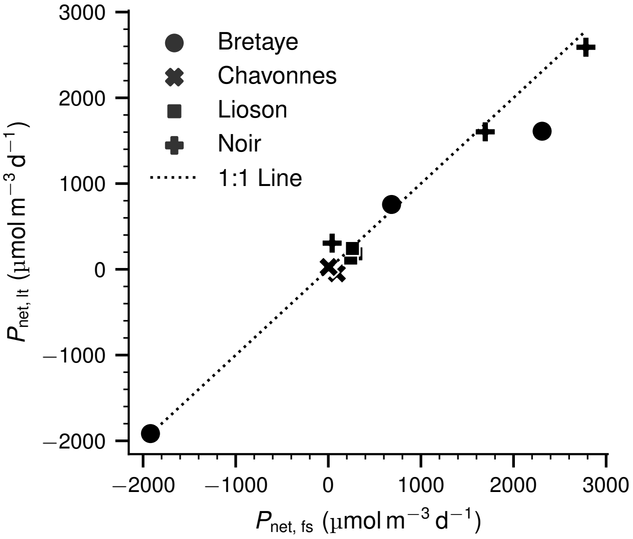

Supplementary Figure 9. Comparison between net CH_4 production rates (P_{net}) rates calculated for the full scale mass balance ($P_{\mathrm{net,fs}}$) and the lateral transport model ($P_{\mathrm{net,lt}}$) for each lake at every sampling date.

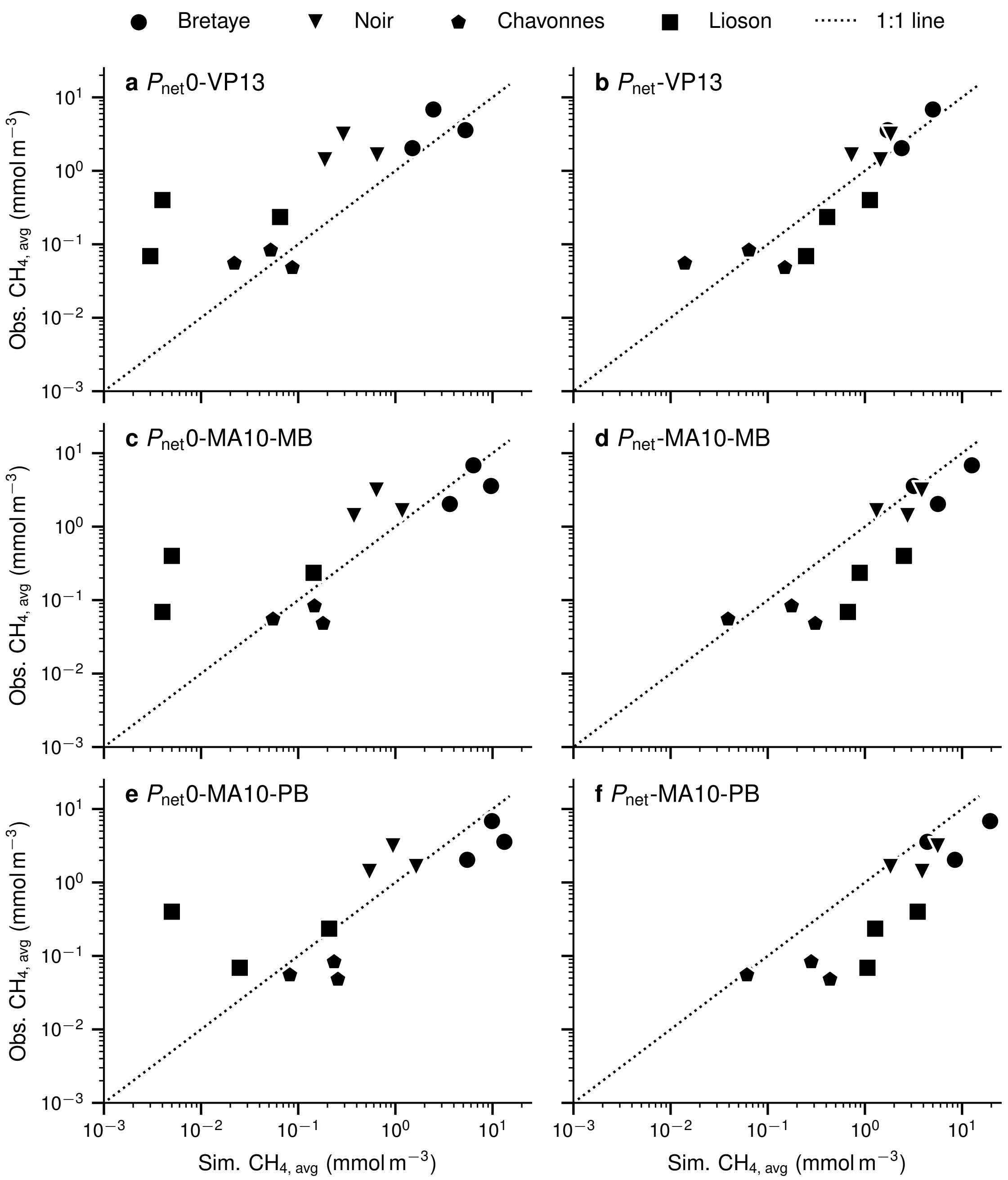

Supplementary Figure 10. Comparison of observed and simulated average surface CH_4 concentration along transects for each campaign. Simulated CH_4 concentrations were obtained with the lateral transport model using k_{600} for diffusive emissions either with (panels **b**, **d** and **f**) or without P_{net} (panels **a**, **c** and **e**). The k_{600} was either estimated by Vachon & Prairie[4] (VP13, panels **a** and **b**), MacIntyre et al.[3] mixed buoyancy (MA10-MB, panels **c** and **d**) and MacIntyre et al.[3] positive buoyancy (MA10-PB, panels **e** and **f**). The statistical results of each panel are shown in Table 2.

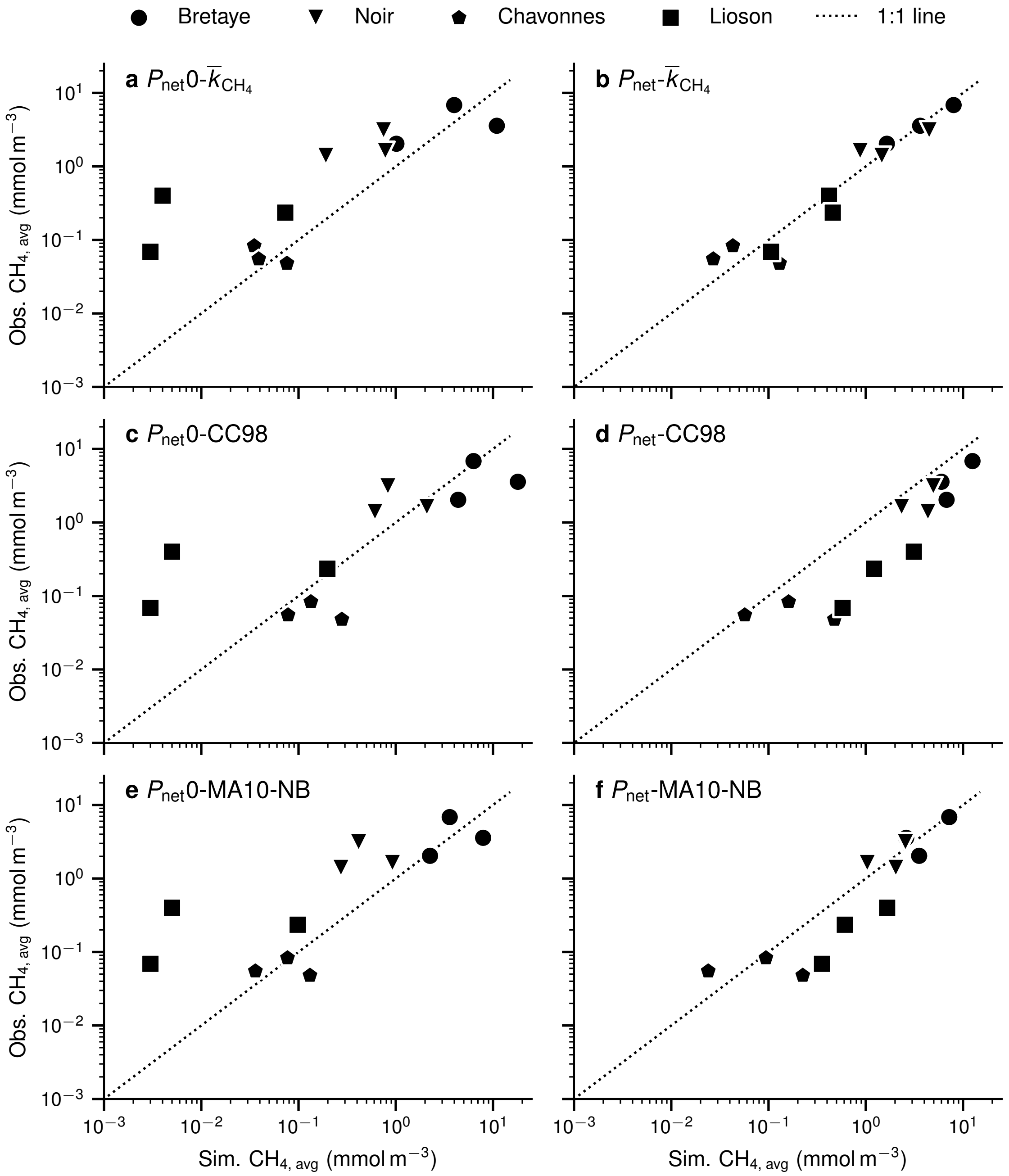

Supplementary Figure 11. Comparison of observed and simulated average surface CH_4 concentration along transects for each campaign. Simulated CH_4 concentrations were obtained with the lateral transport model using k_{600} for diffusive emissions either with (panels **b**, **d** and **f**) or without P_{net} (panels **a**, **c** and **e**). The k_{600} was either the chamber-based k_{600} ($\overline{k}_{CH_4}$, panels **a** and **b**), Cole & Caraco[2] (CC98, panels **c** and **d**) and MacIntyre et al.[3] negative buoyancy (MA10-NB, panels **e** and **f**). The statistical results of each panel are shown in Table 2.

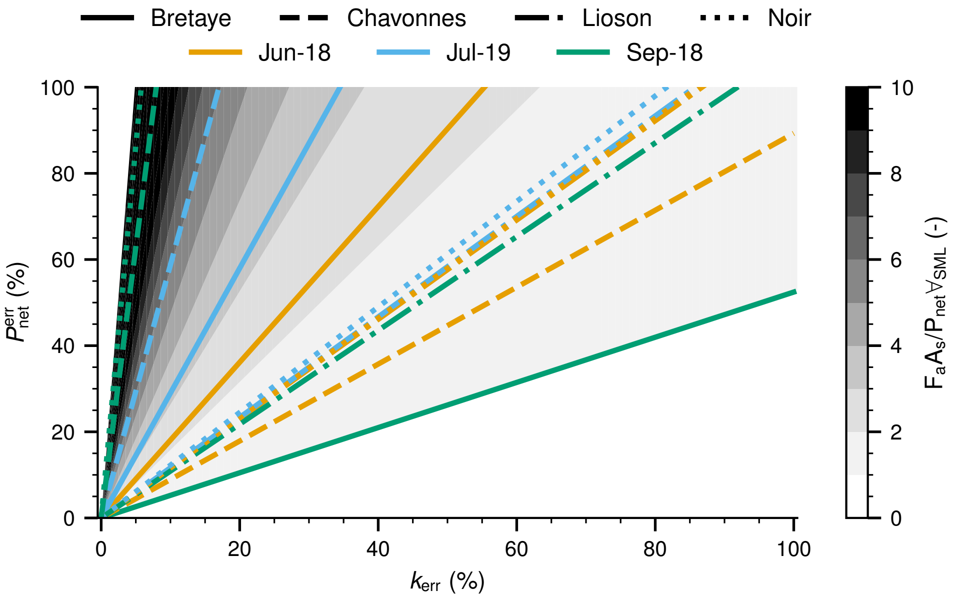

Supplementary Figure 12. Percentage of error on P_{net} calculations (P_{net}^{err}) caused by over- or underestimation of k_{CH_4} (k_{err}) due to the use of k_{600} parameterization. Estimating the ratio between F_aA_a and $P_{net}\forall_{SML}$ from measured values (lines), it is possible to estimate the P_{net} error given the percentage error on k_{CH_4} estimations.

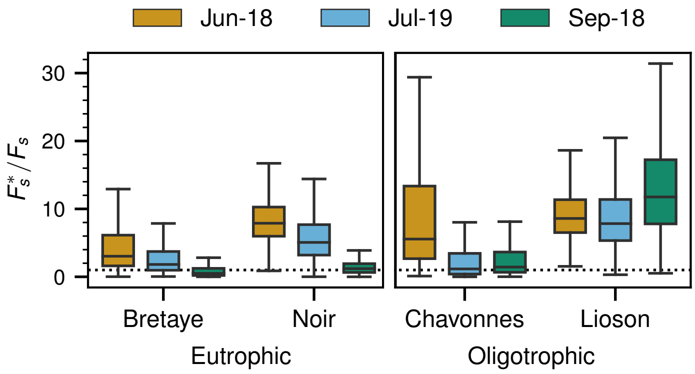

Supplementary Figure 13. Ratio of littoral sediment flux needed (F_s^*) to compensate the surface diffusive emissions compare to measured littoral sediment flux (F_s) considering no net production ($P_{net} = 0$) rate in the surface mixed layer. Boxes show the first and third quartiles with the median (line), whiskers extend to most extreme data point within 1.5 times the interquartile range from the box.

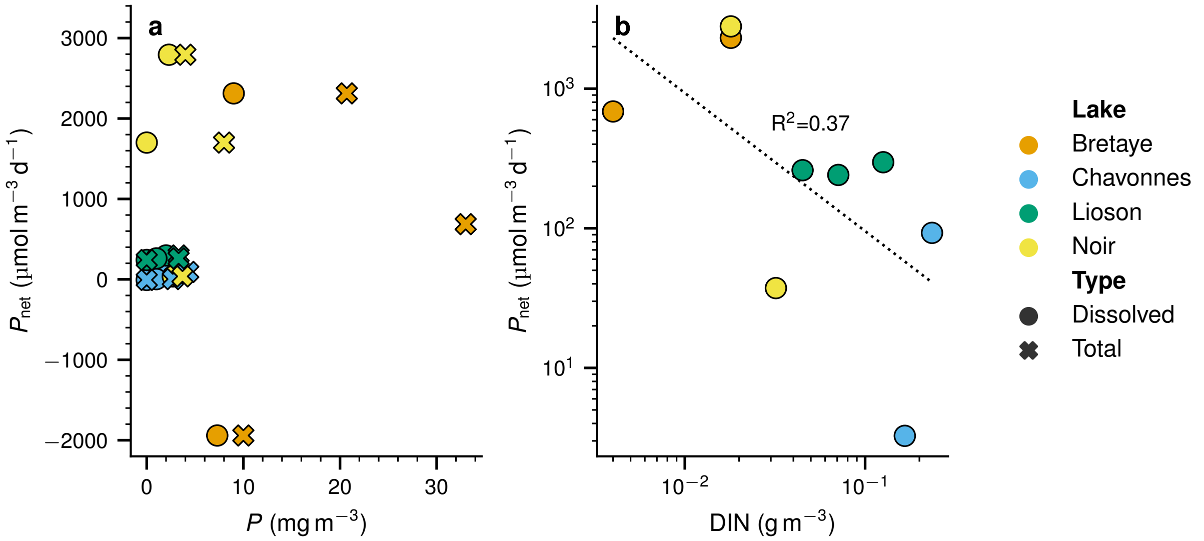

Supplementary Figure 14. **a** Net production rate of CH_4 (P_{net}) rates versus total and dissolved phosphorus concentration (P) and **b** dissolved inorganic nitrogen (DIN) at the surface mixed layer for each lake and sampling campaign.

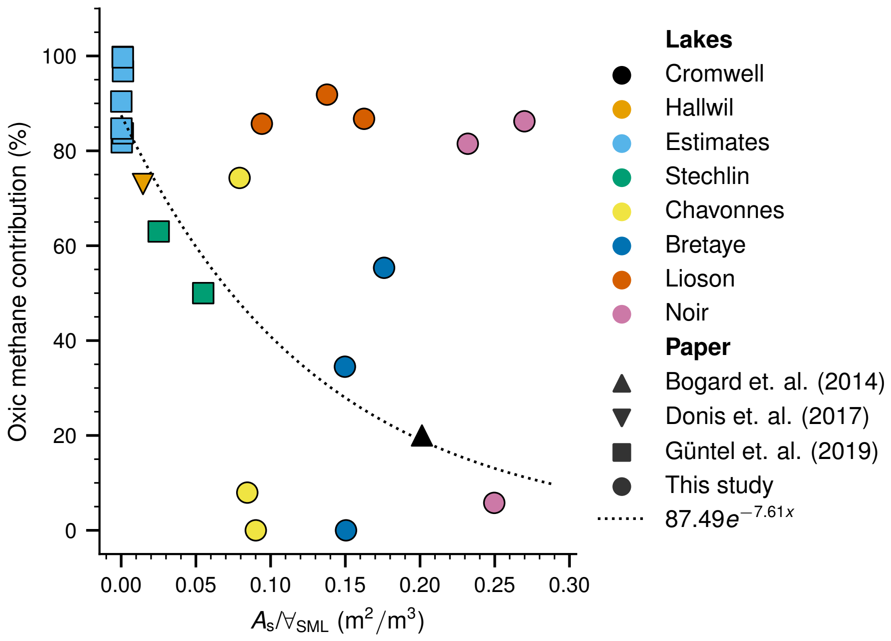

Supplementary Figure 15. Contribution of the net production rate of CH_4 to diffusive emissions (OMC) versus the ratio between the sediment area (A_s) and the epilimnetic volume ($\forall_{SML}$). The trend line $y = 87.49e^{7.61x}$ was proposed by Günthel et al.[6].

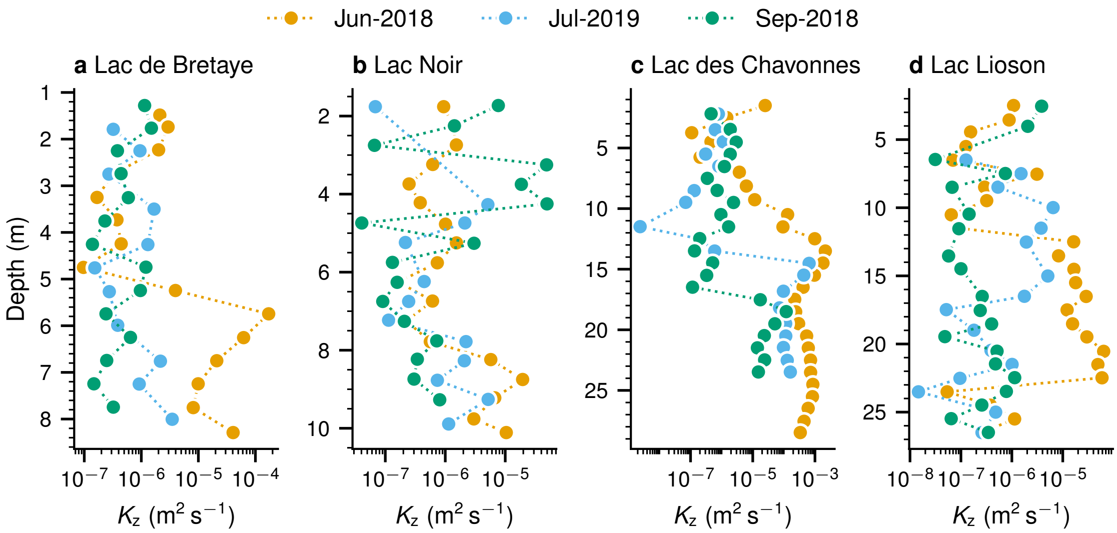

Supplementary Figure 16. Vertical diffusivity (K_z) for **a** Lac de Bretaye, **b** Lac Noir, **c** Lac des Chavonnes and **d** Lac Lioson.

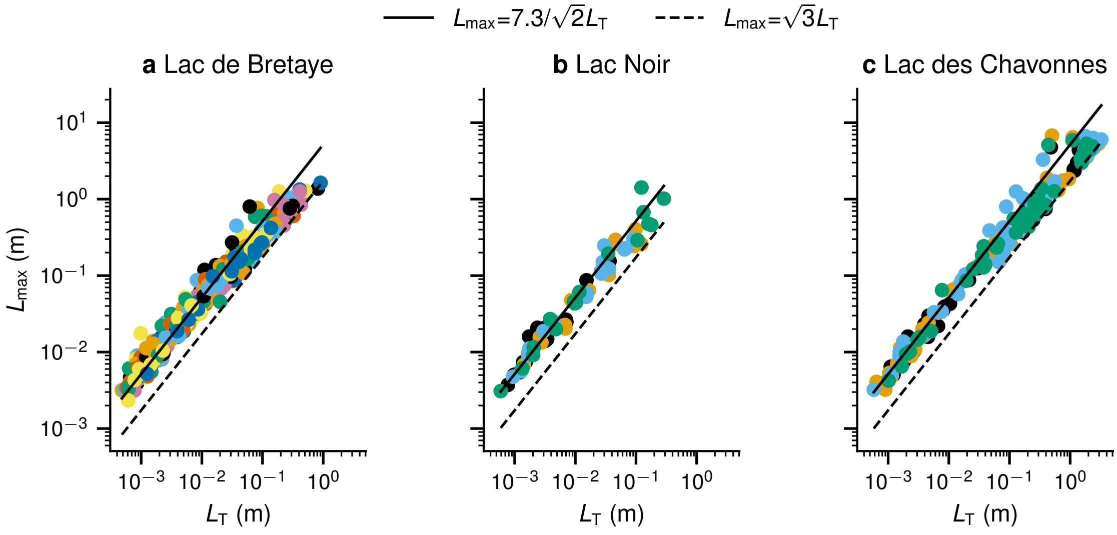

Supplementary Figure 17. Maximum ($L_{\max}$) versus root-mean square (L_{T}) displacement length scales within segments of temperatures microstructure profiles from **a** Lac de Bretaye, **b** Lac Noir and **c** Lac des Chavonnes. Each color represent one profile performed on July 2020.

Supplementary References

[1] Carlson, R. A. Trophic State Index for Lakes. *Limnology And Oceanography.* **22**, 361-369 (1977)

[2] Cole, J. & Caraco, N. Atmospheric Exchange of Carbon Dioxide in a Low-Wind Oligotrophic Lake Measured by the Addition of SF6. *Limnology And Oceanography.* **43**, 647-656 (1998)

[3] MacIntyre, S., Jonsson, A., Jansson, M., Aberg, J., Turney, D. & Miller, S. Buoyancy Flux, Turbulence, and the Gas Transfer Coefficient in a Stratified Lake. *Geophysical Research Letters.* **37**, 2-6 (2010)

[4] Vachon, D. & Prairie, Y. The Ecosystem Size and Shape Dependence of Gas Transfer Velocity versus Wind Speed Relationships in Lakes. *Can. J. Fish. Aquat. Sci.*. **70**, 1-8 (2013)

[5] McGinnis, D. F., Greinert, J., Artemov, Y., Beaubien, S. E. & Wüest, A. Fate of rising methane bubbles in stratified waters: How much methane reaches the atmosphere? *J. Geophys. Res. C: Oceans* **111**, 1–15 (2006).

[6] Günthel, M., Donis, D., Kirillin, G., Ionescu, D., Bizic, M., McGinnis, D., Grossart, H. & Tang, K. Contribution of Oxic Methane Production to Surface Methane Emission in Lakes and Its Global Importance. *Nature Communications.* **10**, 5497 (2019)
